# *Lactobacillus plantarum* LMT1-48 exerts anti-obesity effect in high-fat diet-induced obese mice by regulating expression of lipogenic genes

**DOI:** 10.1038/s41598-020-57615-5

**Published:** 2020-01-21

**Authors:** Woo Jin Choi, Hye Jin Dong, Hyun Uk Jeong, Dong Wook Ryu, Soo Min Song, Yu Ri Kim, Hyun Ho Jung, Tai Hoon Kim, Yeung-Hyen Kim

**Affiliations:** Medytox Gwangkyo R&D center, Medytox Inc., Suwon, Republic of Korea

**Keywords:** Microbiology, Obesity

## Abstract

Obesity is a major health problem and is known to be closely associated with metabolic diseases. Abnormal hepatic accumulation of fat causes fatty liver or hepatic steatosis, and long-term consumption of a high-fat diet is known to be a key obesity-causing factor. Recent studies have demonstrated that probiotics such as *Lactobacillus strains*, exert an anti-obesity effect by regulating adipogenesis. However, it is still unknown how the consumption of probiotics can reduce abdominal fat volume by regulating the hepatic expression of lipogenic genes. Therefore, we evaluated the effect of long-term ingestion of *L*. *plantarum* LMT1-48 on the expression of lipogenic genes in high-fat diet (HFD)-fed mice. We observed that treatment of 3T3-L1 adipocytes with *L*. *plantarum* LMT1-48 extract inhibited their differentiation and lipid accumulation by downregulating lipogenic genes, namely, *PPARγ*, *C/EBPα*, *FAS*, and *FABP4*. Interestingly, administration of *L*. *plantarum* LMT1-48 reduced liver weight and liver triglycerides concurrently with the downregulation of the lipogenic genes *PPARγ*, *HSL*, *SCD-1*, and *FAT/CD36* in the liver, resulting in the reduction of body weight and fat volume in HFD-fed obese mice. Notably, we also observed that the administration of at least 10^6^ CFU of *L*. *plantarum* LMT1-48 significantly lowered body weight and abdominal fat volume in modified diet-fed mouse models. Collectively, these data suggest that *L*. *plantarum* LMT1-48 is a potential healthy food for obese people.

## Introduction

Obesity is a major health problem and causes many diseases, including cardiovascular diseases, type 2 diabetes, and liver diseases^1,2^. In particular, obesity is closely associated with abnormal accumulation of free fatty acids (FFA) in the liver, resulting in fatty liver or hepatic steatosis^3–5^. Fatty liver is classified as microvesicular and macrovesicular fat through the histological accumulation of >5% triglycerides in hepatocytes^6^. The major causes of fatty liver are related to drug use, metabolic syndrome, and alcohol consumption^7,8^. Insulin resistance is the primary cause of metabolic disorders leading to fat accumulation. Insulin-suppressed hormone-sensitive lipases (HSLs) in adipocytes are activated due to insulin resistance, while triglycerides in adipocytes are released into the blood in the form of fatty acids. Free fatty acids released into the blood increase the fatty acid inflow into the liver, whereby hepatic fat accumulation increases. The mechanism underlying the development of nonalcoholic fatty liver is not fully understood yet, but hepatic fat accumulation is considered a feature of insulin resistance with abdominal obesity and is closely related to metabolic syndrome^9,10^.

Consequently, long-term consumption of high-fat enriched diets increases the body weight as well as hepatic fat accumulation and hepatic adipocyte proliferation in mammals, thereby leading to obesity^11,12^. Interestingly, recent studies have demonstrated that human probiotic strains play an important role in modulating immune responses^13,14^ and exert anti-obesity effects in diet-induced obese mice^15^. In particular, *Lactobacillus* species exhibits an anti-obesity effect by regulating the expression of genes related to adipogenesis^16,17^. The expression of lipogenic genes is known to play a pivotal role in obesity-induced metabolic complications. Peroxisome proliferator-activated receptor γ (PPARγ) and CCAAT/enhancer-binding protein α (C/EBPα) are the major activators of adipogenesis^18^, and fatty acid synthase (FAS) and fatty acid-binding protein 4 (FABP4) are involved in the formation of mature adipocytes^19^. Fatty acid translocase (FAT)/CD36 plays an important role in the uptake and oxidation of intracellular long-chain fatty acids^20^. HSL is a key enzyme involved in the mobilization of fatty acids, overall energy homeostasis, and hydrolysis of fatty acids^21^, and stearoyl-CoA desaturase-1 (SCD-1) is the main enzyme involved in the biosynthesis of fatty acids^22^. Thus, lipogenic genes are the key regulators of the synthesis^23^ and accumulation^24^ of fatty acids and differentiation of adipocytes^25^. However, it is still unknown how the ingestion of probiotics can reduce abdominal fat volume by regulating the hepatic expression of lipogenic genes. Therefore, we assessed the functional effects of long-term ingestion of *L*. *plantarum* LMT1-48 by evaluating the expression of lipogenic genes in the livers of high-fat diet (HFD)-fed mice.

## Results

### Inhibitory effect of the cell extract of *L*. *plantarum* LMT1-48 on 3T3-L1 adipocytes

**Figure 1:**
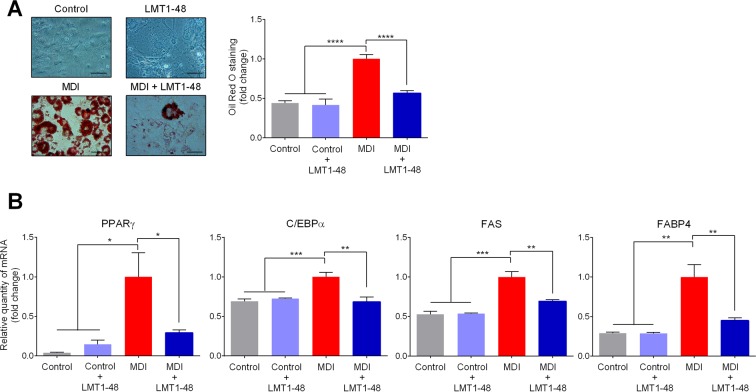
Inhibitory effect of *L*. *plantarum* LMT1-48 in 3T3-L1 adipocytes. (**A**) 3T3-L1 cells were stimulated with MDI reagent for 48 h to induce differentiation. These cells were then treated with the *L*. *plantarum* LMT1-48 extract (final concentration of 200 µg/ml). After differentiation, 3T3-L1 cells were stained with Oil red O reagent. To quantitate the inhibitory effect of *L*. *plantarum* LMT1-48 on the differentiation, Oil Red O stained fat droplets were dissolved in isopropanol, and the amount of the dissolved stain was spectrophotometrically measured at 520 nm by an ELISA reader. (**B**) Meanwhile, the mRNA levels of the lipogenic genes *PPARγ*, *C/EBPα*, *FAS*, and *FABP4* in differentiated 3T3-L1 cells were quantitated. Two independent experiments were performed with n = 5 for each experiment. Error bars represent standard error of the mean (Mean ± SEM). *P < 0.05, **P < 0.01, ***P < 0.001.

Obesity is associated with an increase in adipocyte number and size^25^, and activation of adipogenesis factors^26^. Therefore, we investigated the mechanism by which adipocytes were regulated in response to treatment with *L*. *plantarum* LMT1-48. For this, we used 3T3-L1 cells and differentiated them into adipocytes by treating them with MDI reagent. Cellular viability was evaluated to assess for the toxicity of the *L*. *plantarum* LMT1-48 extract, and no harmful effect of the extract on the cellular viability was detected (Supple. Fig. S1A,B). The differentiation of 3T3-L1 cells was significantly inhibited in the presence of the *L*. *plantarum* LMT1-48 extract (Fig. 1A, Left panel of the staining data). Oil Red O staining was carried out to assess for the inhibitory effect of *L*. *plantarum* LMT1-48 and the results were compared with those of the MDI-treated control group (Fig. 1A, Right panel).

Further, we used quantitative RT-PCR to assess whether the *L*. *plantarum* LMT1-48 extract altered the expression of the genes related to adipocyte differentiation. Among these genes, *PPARγ*, *C/EBPα*, *FAS*, and *FABP4*, were significantly upregulated in the presence of MDI; however, they were downregulated in response to the treatment with the *L*. *plantarum* LMT1-48 extract (Fig. 1B). These results indicate that the *L*. *plantarum* LMT1-48 extract inhibited the differentiation of and lipid accumulation in 3T3-L1 adipocytes by downregulating lipogenic genes.

### Anti-obesity effect of *L*. *plantarum* LMT1-48 in HFD-fed mice

**Figure 2:**
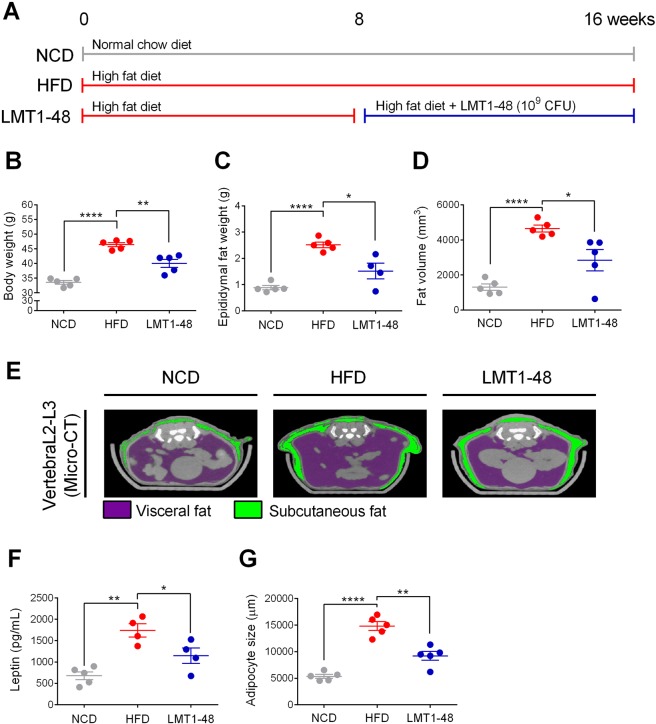
Anti-obesity effect of *L*. *plantarum* LMT1-48 in HFD-fed mice. (**A**) Experimental scheme. (**B**) Body weight, (**C**) epididymal fat weight, and (**D**) fat volume were measured to assess for the anti-obesity effect of *L*. *plantarum* LMT1-48 in mice. (**E**) Representative micro-computed tomography (CT) images of the abdominal fat at L2–L3 region. The visceral and subcutaneous fats are depicted in purple and green colors, respectively. (**F**) Serum levels of the leptin hormone were measured via ELISA (**G**) The adipocyte size in the visceral adipose tissue was measured. Two independent experiments were performed with n = 5 for each experiment. Error bars represent standard error of the mean (Mean ± SEM). *P < 0.05, **P < 0.01, ***P < 0.001, ****P < 0.0001.

To assess for the anti-obesity effect of *L*. *plantarum* LMT1-48 in HFD-fed mice, we designed three different feeding conditions as shown in Fig. 2A. Long-term HFD feeding increased the body weights of mice (46.4 ± 1.6 g) compared with those of the NCD-fed mice (33.5 ± 1.4 g). Administration of *L*. *plantarum* LMT1-48 reduced the body weights significantly (40.1 ± 3.0 g) compared with those of the HFD-fed mice (Fig. 2B).

We also assessed for changes in the epididymal fat pad weight and abdominal fat volume using a micro-CT scanning system. Administration of *L*. *plantarum* LMT1-48 significantly decreased the epididymal fat pad weight (Fig. 2C) and abdominal fat volume in HFD-fed mice (Fig. 2D). Representative positron emission tomography (PET/CT) scanning images are shown in Fig. 2E, where visceral and subcutaneous fat are depicted in purple and green colors, respectively (Fig. 2E).

Recent studies have shown that the leptin hormone is secreted from the adipose tissue and is highly associated with obesity^27–29^. Therefore, we evaluated the plasma levels of leptin and found that LMT1-48 group had lower levels than the HFD group (Fig. 2F). We also measured the size of visceral adipocytes to assess for the anti-obesity effect of *L*. *plantarum* LMT1-48 in HFD-fed mice. Histological analysis showed that adipocyte size in the visceral adipose tissue was smaller in LMT1-48 group than in the HFD group (Fig. 2G). These results indicate that administration of *L*. *plantarum* LMT1-48 exerts an anti-obesity effect by downregulating plasma levels of leptin, resulting in inhibition of fat accumulation and adipocyte proliferation in HFD-fed mice.

### *L*. *plantarum* LMT1-48 downregulates lipogenic genes in the livers of HFD-fed mice

**Figure 3:**
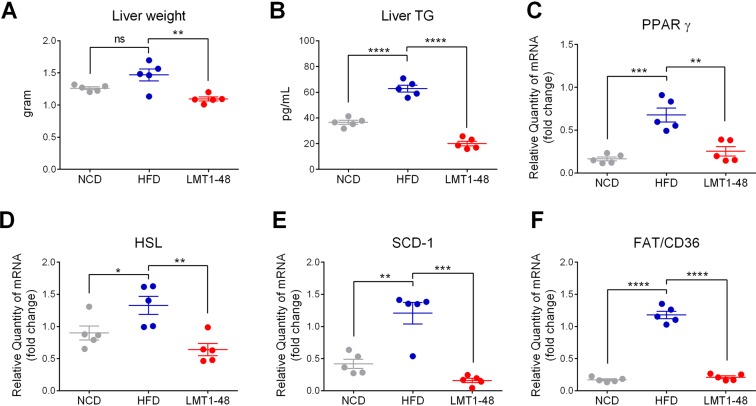
Effect of *L*. *plantarum* LMT1-48 on the expression of lipogenic genes in the liver of HFD-fed mice. (**A**) Liver weight and (**B**) liver triglycerides were measured to assess the influence of *L*. *plantarum* LMT1-48 administration in HFD-fed mice (n = 5 mice per group). (**C**–**F**) Hepatic mRNA levels of the lipogenic genes (**C**) *PPARγ*, (**D**) *HSL*, (**E**) *SCD*-1, and (**F**) *FAT/CD36* were measured by quantitative real-time PCR. Two independent experiments were performed with n = 5 for each experiment. Error bars represent standard error of the mean (Mean ± SEM). *P < 0.05, **P < 0.01, ***P < 0.001, ****P < 0.0001.

We assessed the expression of lipogenic genes in the livers of HFD-fed mice. Administration of *L*. *plantarum* LMT1-48 significantly reduced the liver weight (Fig. 3A), liver triglycerides (Fig. 3B), and expression of the lipogenic genes PPARγ (0.2 ± 0.1 in NCD group, 0.7 ± 0.2 in HFD group, and 0.3 ± 0.1 in LMT1-48 group), HSL (0.9 ± 0.2 in NCD group, 1.3 ± 0.3 in HFD group, and 0.6 ± 0.2 in LMT1-48 group), SCD1 (0.4 ± 0.2 in NCD group, 1.2 ± 0.4 in HFD group, and 0.2 ± 0.1 in LMT1-48 group), and FAT/CD36 (0.2 ± 0.0 in NCD group, 1.2 ± 0.1 in HFD group, and 0.2 ± 0.1 in LMT1-48 group) in the livers of HFD-fed mice (Fig. 3C–F). These results indicate that administration of *L*. *plantarum* LMT1-48 has an anti-obesity effect by reducing liver weight and liver triglycerides, and downregulating the expression of lipogenic genes in the livers of HFD-fed obese mice.

### Anti-obesity effects of *L*. *plantarum* LMT1-48 on modified-diet–fed mice

**Figure 4:**
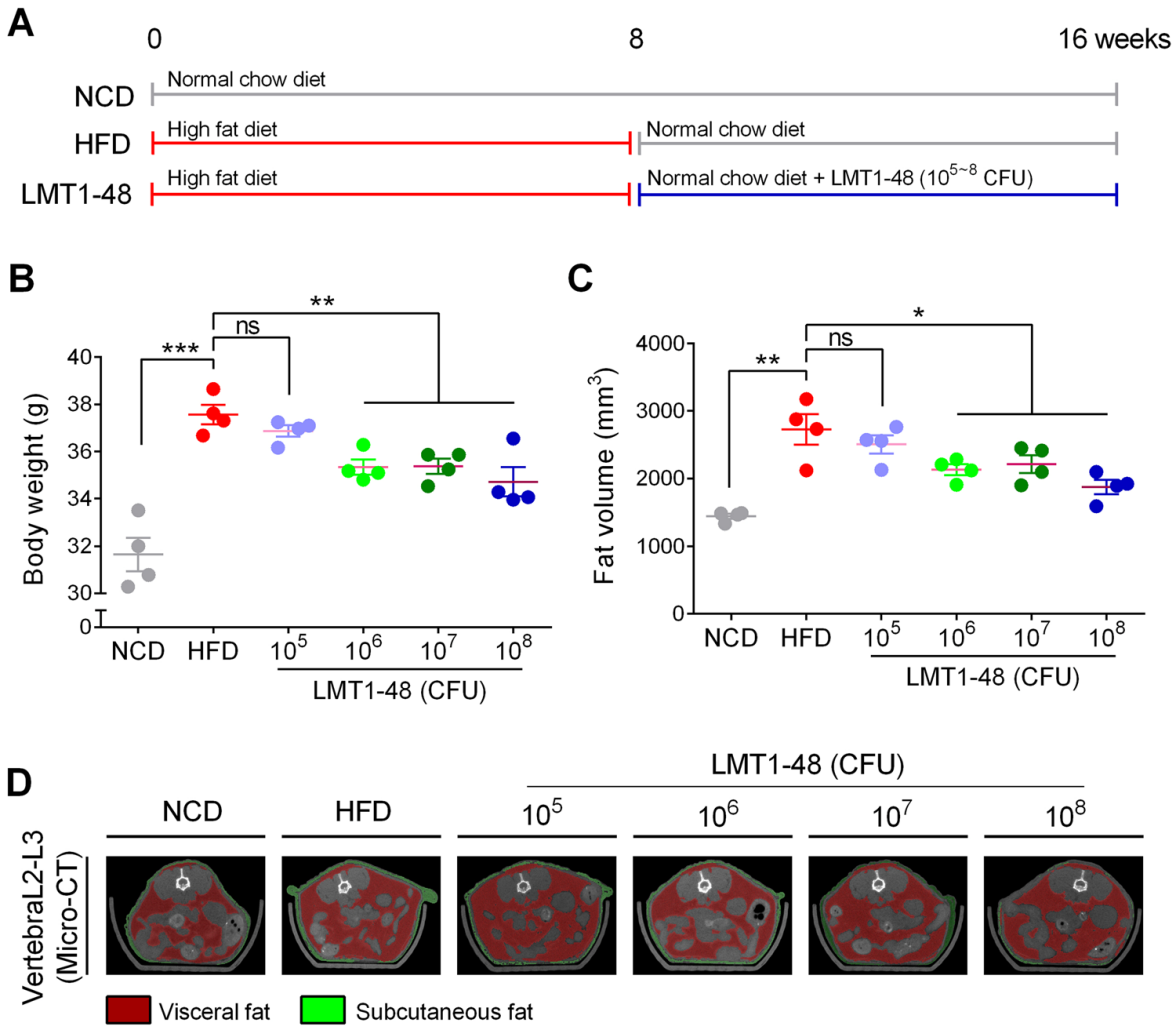
Anti-obesity effect of *L*. *plantarum* LMT1-48 in modified-HFD–fed mice. (**A**) Experimental scheme. (**B**) Body weight and (**C**) fat volume were measured to assess the anti-obesity effect of different concentrations (10^5^, 10^6^, 10^7^, or 10^8^ CFU) of the *L*. *plantarum* LMT1-48 extract in modified-HFD–fed mice (*n* = 4 mice per group). (**D**) Representative micro-computed tomography (CT) image of the abdominal fat (L2–L3 region). The visceral and subcutaneous fats are depicted in red and green colors, respectively. Two independent experiments were performed with n = 4 for each experiment. Error bars represent standard error of the mean (Mean ± SEM). *P < 0.05, **P < 0.01, ***P < 0.001.

To assess whether the anti-obesity effect of the *L*. *plantarum* LMT1-48 extract was dose-dependent, modified-diet–fed mice were administered with different doses of the extract (10^5^, 10^6^, 10^7^, or 10^8^ CFU) per mouse. We designed six different feeding conditions as shown in Fig. 4A. Long-term feeding of HFD followed by normal chow diet led to an increase in body weight in HFD group (37.6 ± 0.8 g) compared with that in the NCD group (31.6 ± 1.4 g, Fig. 4B). Administration of at least 10^6^ CFU of *L*. *plantarum* LMT1-48 significantly lowered the body weight (10^6^ CFU: 35.3 ± 0.7 g; 10^7^ CFU: 35.4 ± 0.6 g; 10^8^ CFU: 34.7 ± 1.2 g) in LMT1-48 group, whereas this response was not observed in mice administered with 10^5^ CFU of *L*. *plantarum* LMT1-48 (36.9 ± 0.5 g; Fig. 4B). Therefore, we further measured fat volume using a micro-CT scanning system to assess for changes in abdominal fat volume in diet-induced obese mice. Similar to body weight changes, administration of at least 10^6^ CFU of *L*. *plantarum* LMT1-48 significantly reduced abdominal fat volume in HFD-fed mice (Fig. 4C). A representative PET/CT image is provided in Fig. 4D, with visceral and subcutaneous fats depicted in red and green colors, respectively. These results indicate that an appropriate amount of the *L*. *plantarum* LMT1-48 extract is required to have an anti-obesity effect in HFD-fed mice.

## Discussion

Obesity is closely associated with liver abnormalities, including nonalcoholic fatty liver disease (NAFLD) and steatohepatitis^2,30^. Fat accumulation in the liver is generally caused by increased lipotoxicity resulting from high levels of free fatty acids, free cholesterol, and lipid metabolites^31^, and these, in turn, are regulated by lipogenic genes, such as PPARγ, C/EBPα, FAS, and FABP4^18,19^. Importantly, these lipogenic genes play an important role in obesity-induced metabolic liver diseases^19^. Additionally, several studies have also demonstrated that liver weight, liver triglycerides, and expression of lipogenic genes are closely associated with obesity^2,30^, and the administration of lactic acid bacteria (LAB) inhibits the synthesis^32^ and accumulation^33^ of fats in HFD-induced obese mice. Therefore, we hypothesized that the administration of *L*. *plantarum* LMT1-48 could reduce body fat by regulating the expression of lipogenic genes in the livers of HFD-induced obese mice.

Notably, recent studies have demonstrated that a high amount of lipopolysaccharides (LPS) produced by gram-negative bacteria cause chronic inflammation, and obese people have been reported to have higher levels of gram-negative bacteria in their gastrointestinal tracts^34,35^. Importantly, LPS is known to leak through damaged intestinal epithelial barrier under inflammatory conditions^36^ and circulate to the liver through the portal vein. This is accompanied by an increase in the levels of PPARγ^37–39^, causing obesity and metabolic diseases via an inflammatory immune response^40^. Importantly, the hepatic expression of PPARγ is highly associated with NAFLD in humans and experimental mouse models^41^. Moreover, fat accumulation in hepatocytes of nonalcoholic fatty liver is closely related to insulin resistance, and obesity and insulin resistance are also correlated with NAFLD^9,10^. In general, PPARγ is expressed at very low levels in the liver; however, insulin upregulates the expression of PPAR-γ in the adipose tissue, leading to increased adipogenesis and lipogenesis in the liver during obesity^42,43^. Although HSL produces fatty acids and glycerol by breaking down triglycerides in the adipose tissue and thereby inhibits fat accumulation^44^, increased insulin resistance in obese patients reduces HSL levels and inhibits lipolysis activation. Interestingly, in the present study, we observed that the administration of *L*. *plantarum* LMT1-48 reduced liver weight and liver triglycerides concurrently with the downregulation of PPARγ and HSL in the liver, resulting in the reduction of body weight and fat volume in HFD-fed mice. Therefore, we speculate that the administration of *L*. *plantarum* LMT1-48 may regulate LPS levels along with downregulation of PPARγ and HSL, resulting in reduced body fat and liver weight in HFD-induced obese mice.

Several studies have also demonstrated that short-chain fatty acids (SCFAs) produced by LAB exert an anti-obesity effect through the modulation of the lipid and glucose metabolisms^45,46^, resulting in reduced adipocyte size^47,48^. Notably, SCFAs play a potential role in decreasing fat accumulation in the adipose tissue by accelerating the oxidation of fatty acids in HFD-induced obese mice^49,50^. SCFAs can switch the metabolic state from lipogenesis to fat oxidation by regulating the expression of PPARγ in HFD-induced obese rodents^51,52^. Interestingly, in the present study, we also observed that treatment with the *L*. *plantarum* LMT1-48 extract inhibited adipocyte differentiation and lipid accumulation and downregulated the lipogenic gene PPARγ. However, the mechanism underlying the SCFA-mediated regulation of the expression of the lipogenic genes HSL, SCD-1, and FAT/CD36 has not been clearly studied yet. Based on the present study, we hypothesize that administration of *L*. *plantarum* LMT1-48 may help produce certain physiologically active substances, such as SCFAs, which are known to regulate lipid accumulation and adipocyte differentiation through downregulation of lipogenic genes. To address this, we need to further investigate the mechanism through which SCFAs produced by *L*. *plantarum* LMT1-48 regulate fat accumulation and differentiation of adipocytes in HFD-induced obese mice.

Recent studies have shown that the amount of LAB is important to maintain the colonization and habitation in the gut^53,54^, and thus LAB dosage may influence the effectivity of the treatment on obesity^55^. In particular, most LAB studies have evaluated the efficacy at the dosage of 10^9^ CFU^56,57^. The reason is that this amount is the maximum viable count of LAB that can be grown^58^. Therefore, in this study, we evaluated the anti-obesity effect at this dosage and compared the results with those derived from lower doses (10^5^~10^8^ CFU). Interestingly, we observed that the administration of *L*. *plantarum* LMT1-48 showed the anti-obesity effect when used at a dosage of >10^6^ CFU but not at 10^5^ CFU. Although further investigation is needed to understand the role of colonization in the LAB-induced anti-obesity effect, we assume that 10^6^ CFU is the minimum dose of colonization in the gut for *L*. *plantarum* LMT1-48 to induce the anti-obesity effect.

To summarize, this is the first study that has demonstrated that *L*. *plantarum* LMT1-48 exerts an anti-obesity effect in HFD-fed mice through downregulation of the hepatic expression of lipogenic genes along with the consequent reduction in the number of adipocytes. Although further studies are needed to elucidate whether *L*. *plantarum* LMT1-48 regulates the body fat mass in humans, our results offer *L*. *plantarum* LMT1-48 as a potential probiotic for obese people.

## Materials and Methods

### Assessment of the inhibitory effect of *L*. *plantarum* LMT1-48 on 3T3-L1 adipocytes

3T3-L1 cells (KCBL 10092.1) were obtained from Korean cell line bank (KCBL, Seoul, Korea) and sub-cultured up to 10 passages in Dulbecco’s modified Eagle medium (DMEM, Gibco, NY, USA) containing 10% fetal bovine serum (FBS, Invitrogen, Auckland, New Zealand) and 1% penicillin/streptomycin (P/S, Gibco, NY, USA) at 37 °C in a humidified incubator with 5% CO_2_. 3T3-L1 cells were seeded at a density of 6 × 10^4^ cells/well in 12-well plates and cultured for 48 h at 37 °C. For adipocyte differentiation, cells were stimulated with MDI (3-Isobutyl-1-methylxanthine, dexamethasone, and insulin) reagent for 48 h at 37 °C. MDI reagent consisted of 0.5 mM 3-isobutyl-1-methylxanthine (IBMX, Sigma, St. Louis, MO, USA), 0.25 nM dexamethasone (Sigma, St. Louis, MO, USA), and 5 μg/mL insulin (Sigma, St. Louis, MO, USA). MDI-stimulated cells were cultured in DMEM containing 10% FBS and 5 μg/mL insulin for 4 days at 37 °C.

To test the inhibitory effect of *L*. *plantarum* LMT1-48 on 3T3-L1 adipocytes, the cell extract of *L*. *plantarum* LMT1-48 was prepared. Briefly, *L*. *plantarum* LMT1-48 strain (KCTC registered identification number: KCTC13024BP; patent registration number: KR101670048B1; GenBank accession number: LC131441.1) was isolated from the traditional Korean fermented food kimchi and cultured in De Man, Rogosa, and Sharpe (MRS; Difco Co., MI, USA) broth for 24 h at 37 °C. Cultured cells were centrifuged at 9358 × *g* at 4 °C for 10 min and washed thrice with PBS. Cells were mechanically lysed using a beat beater (Bullet Blender, Next Advance, NY, USA) for 3 min, and samples were then placed on ice for another 3 min. The procedure following the lysis and PBS washes were repeated five times. Cell debris was removed by centrifugation at 9358 × *g* for 5 min at 4 °C, and the supernatant was filtered through a Microcon filter with 0.45-µm pores under sterilized conditions. MDI-treated 3T3-L1 adipocytes were then treated with this cell extract at a final concentration of 200 µg/mL.

### Cellular viability assay

The viability test for 3T3-L1 cells was performed using Cell Counting Kit-8 (CCK-8, Dojindo Laboratories, Kumamoto, Japan). In brief, 3T3-L1 cells were placed at a density of 5 × 10^3^ cells/well in 96-well plates and cultured for 24 h at 37 °C. Subsequently, the cells were further cultured with *L*. *plantarum* LMT1-48 extract alone (25, 50, 100, 200, 400, or 800 μg/mL) or with a combination of *L*. *plantarum* LMT1-48 extract (200 µg/mL) and MDI reagent for 48 h at 37 °C. The CCK-8 reagent (10 μL) was administered to the cells, which were then incubated for another 2 h at 37 °C. The absorbance was measured at 450 nm using an ELISA reader (Biotek, VT, USA).

### Oil red O staining

After assessing the inhibitory effect of *L*. *plantarum* LMT1-48 on 3T3-L1 adipocytes, we assessed its effect on the adipogenic differentiation by Oil red O staining. Briefly, cells were washed twice with PBS and fixed with 10% formaldehyde (Merck, NJ, USA) for 5 min at room temperature. Afterward, cells were treated with fresh 10% formaldehyde for another 1 h at room temperature. Subsequently, formaldehyde was removed and cells were stained with Oil red O (Sigma, St. Louis, MO, USA) for 1 h at room temperature. Next, Oil red O solution was removed and 3T3-L1 adipocytes were washed 4 times with dH_2_O. Images of stained fat droplets were captured using a microscope from Olympus (Tokyo, Japan). For quantitation, stained fat droplets were dissolved in 1 ml of 100% isopropanol for 10 min at room temperature, and the absorbance was measured at 520 nm using an ELISA reader (Biotek, VT, USA).

### Animal models

Male C57BL/6N mice were purchased from OrientBio Inc. (Seongnam-si, Korea). All the mice were maintained at a specialized pathogen-free facility at CHA University (Seongnam-si, Korea) and used at 5 weeks of age for the experiments. All the animal experiments were performed according to the protocol approved by the Institutional Animal Care and Use Committee of CHA University (IACUC170127).

We designed three different treatment groups based on diet-induced obesity and used at least 5 mice per group. Mice in the negative control group (NCD group) were fed with a normal chow diet (Teklad global certified and irradiated 18% protein rodent diet, 2918 C, Envigo, UK) for 16 weeks. Mice in the positive control group (HFD group) were fed with an HFD (DIO rodent purified diet with 45% energy from fat-red, 58V8, Test Diet, UK) for 16 weeks. To evaluate the anti-obesity effect of *L*. *plantarum* LMT1-48, mice were fed with an HFD for 8 weeks and then administered *L*. *plantarum* LMT1-48 (10^9^ CFU/day) concurrently with HFD for another 8 weeks (LMT1-48 group).

Further, we designed six different modified diet-fed obese animal models. Mice in the negative control group were treated as described above. Mice in the positive control group (HFD group) were fed with HFD for 8 weeks and then supplied with a normal chow diet for another 8 weeks. To evaluate the anti-obesity effect of *L*. *plantarum* LMT1-48, mice were fed with HFD for 8 weeks and then administrated *L*. *Plantarum* LMT1-48 (10^5^, 10^6^, 10^7^, or 10^8^ CFU/day) concurrently with HFD for another 8 weeks (LMT1-48 group). *L*. *plantarum* LMT1-48 strain was freshly prepared and orally administered every day. After 16 weeks, mice were sacrificed, and the visceral fat and liver were collected to assess the adipocyte size and expression of lipogenic genes.

### Micro-computed tomography (CT) analysis

The body fat mass of 5 mice from each group was analyzed with a micro-imaging system (NFR Polaris G90, NanoFocusRay co. Ltd., Suwon-si, Korea). Three-dimensional (3D) images were reconstructed using the 3D analysis software Amira 5.4.1 (Visage Imaging GmbH, Germany). Adipose tissue regions were drawn on the abdominal subcutaneous and visceral fat tissues at the level of the vertebra (L2–L3 region).

### Measurement of adipocyte size

The visceral fat tissues were collected from the experimental animals and fixed in 10% neutral buffered formalin for 48 h. The samples were dehydrated before embedding in paraffin wax and then sectioned with a thickness of 4 μm. Sections were mounted on slides for hematoxylin and eosin (H&E) staining. Digital images were captured at 100× magnification using an inverted microscope (Olympus, Tokyo, Japan), and images were analyzed using Image J software (http://imagej.nih.gov/ij/).

### Measurement of leptin hormone in mouse serum

To analyze hormone levels in sera, blood was collected from the caudal vena cava using a clot activator-coated tube and centrifuged at 2,000 × *g* for 15 min at 4 °C. Leptin levels in sera were determined using a mouse leptin enzyme-linked immunosorbent assay (ELISA) kit (Abcam Inc., Cambridge, UK).

### Measurement of triglyceride in mouse liver

To analyze triglyceride levels in liver, liver tissues was homogenized in lysis buffer containing 5% NP-40 (BioVision, CA, USA) by using a tissue homogenizer. Triglycerides were measured with the Triglyceride Quantification Colorimetric/Fluorometric Kit (BioVision, CA, USA), following the manufacturer’s instructions.

### Quantitative real-time PCR

To quantitate the expression of lipogenic genes in 3T3-L1 adipocytes, total RNA was isolated from cultured 3T3-L1 cells using the TRIzol reagent (Invitrogen, CA, USA), and 50 ng of total RNA was used for cDNA synthesis using RocketScript™ Cycle RT PreMix (Bioneer, Daejeon, Korea). Gene expression was detected using SYBR green (Takara, Tokyo, Japan) on a CFX96 real-time system (Bio-rad, CA, USA). The primers used were as follows: *PPARγ*, forward 5′-GCATGGTGCCTTCGCTGA-3′ and reverse 5′-TGGCATCTCTGTGTCAACCATG-3′; C/*EBPα*, forward 5′-CAAGAACAGCAACGAGTACCG-3′ and reverse 5′-GTCACTGGTCAACTCCAGCAC-3′, *FAS*, forward 5′-TGGGTTCTAGCCAGCAGAGT-3′ and reverse 5′-ACCACCAGAGACCGTTATGC-3′; and *FABP4*, forward 5′-AGTGGGCTTTGCCACAA-3′ and reverse 5′-GGTGATTTCATCGAATTCCA-3′. The expression levels of target genes were normalized to that of *GAPDH* (forward 5′-AACGACCCCTTCATTGAC-3′ and reverse 5′-TCCACGACATACTCAGCAC-3′).

To assess the expression levels of lipogenic genes in the liver, total RNA was isolated from the liver using the TRIzol reagent. Samples were homogenized using a homogenizer (Biomasher, Tokyo, Japan), and cDNA was synthesized from 1 μg of total RNA using RocketScript™ Cycle RT PreMix. Gene expression was detected using SYBR green on a CFX96 real-time system. The primers used were as follows: *PPARγ*, forward 5′-GCATGGTGCCTTCGCTGA-3′ and reverse 5′-TGGCATCTCTGTGTCAACCATG-3′; *HSL*, forward 5′-GGCTCACAGTTACCATCTCACC-3′ and reverse 5′-GAGTACCTTGCTGTCCTGTCC-3′; SCD-1, forward 5′-TGGGTTGGCTGCTTGTG-3′ and reverse 5′-GCGTGGGCAGGATGAAG-3′; and *FAT/CD36*, forward 5′-GATGTGGAACCCATAACTGGATTCAC-3′ and reverse 5′-GGTCCCAGTCTCATTTAGCCACAGTA-3′. Expression of *GAPDH* (forward 5′-GAGACCTTCAACACCCC-3′ and reverse 5′-GTGGTGGTGAAGCTGTAGCC-3′) was used as an internal control. The inverse log of ΔΔCT was then calculated.

### Statistical analysis

Data from all the experiments were analyzed using Prism software (Prism version 7; GraphPad Software, San Diego, CA). An unpaired Student’s *t*-test was used to compare the means of the experimental groups. A *P*-value < 0.05 was considered statistically significant.

## Supplementary information

Supplementary Information
