# Supplementary material for: Lactobacillus plantarum LMT1-48 exerts anti-obesity effect in high-fat diet-induced obese mice by regulating expression of lipogenic genes
Source: Sci Rep. 2020 Jan 21;10:869. doi: 10.1038/s41598-020-57615-5 (PMC6972779; doi:10.1038/s41598-020-57615-5)

***Lactobacillus plantarum* LMT1-48 exerts anti-obesity effect in high-fat diet-induced obese mice by regulating expression of lipogenic genes**

Woo Jin Choi, Hye Jin Dong, Hyun Uk Jeong, Dong Wook Ryu, Soo Min Song, Yu Ri Kim, Hyun Ho Jung, Tai Hoon Kim,* and Yeung-Hyen Kim*

Medytox Gwangkyo R&D center, Medytox Inc., Suwon, Republic of Korea.

Running title: Anti-obesity effect of *Lactobacillus plantarum* LMT1-48

*Y. -H. K. and T. H. K. are joint senior authors.

Corresponding author:

Address for correspondence and reprint requests: Yeung-Hyen Kim, Ph.D., Medytox Gwangkyo R&D center, Medytox Inc., 114 Central town-ro, Yeongtong-gu, Suwon-si, Gyeonggi-do, 16506, Republic of Korea.

Phone: +82 31 8065 8104; Fax: +82 31 8065 8232, E-mail: yhkim@medytox.com or yhkim61@gmail.com

Supplementary figure legend

Supplementary Fig. S1. The effect of the *L. plantarum* LMT1-48 extract on the viability of 3T3-L1 adipocytes

(A) The viability of 3T3-L1 cells under various concentrations of the *L. plantarum* LMT1-48 extract (0, 25, 50, 100, 200, 400, or 800 μg/ml) was evaluated. (B) The *L. plantarum* LMT1-48 extract (200 μg/ml) was administered to 3T3-L1 cells alone or in combination with MDI reagent to assess the viability of 3T3-L1 cells undergoing adipogenic differentiation. The absorbance was measured at 450 nm using an ELISA reader. Two independent experiments were performed with n = 5 for each experiment. Error bars represent standard error of the mean (Mean ± SEM). ****P < .0001.

Supplementary Fig. S1

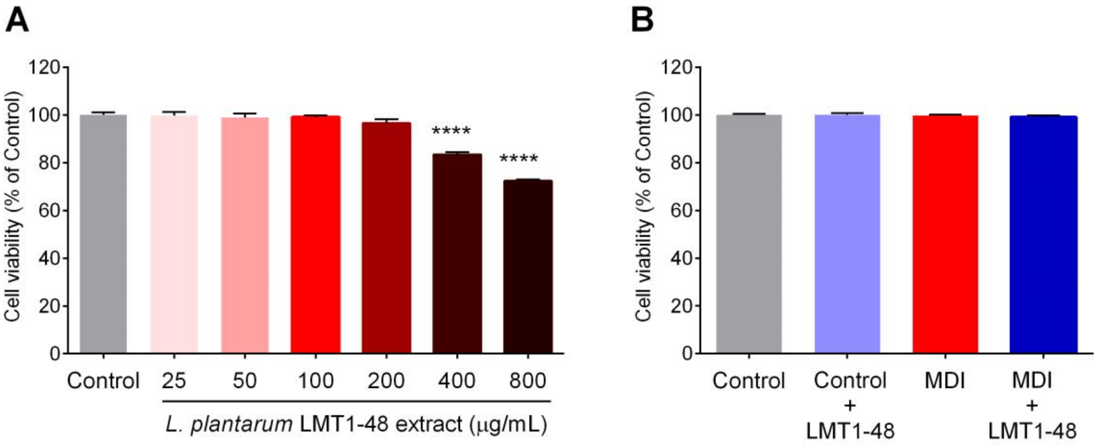

Supplement: Supplementary file 1 — Supplementary Information [file 41598_2020_57615_MOESM1_ESM.pdf]
